# Multiple Sclerosis Risk Allele in *CLEC16A* Acts as an Expression Quantitative Trait Locus for *CLEC16A* and *SOCS1* in CD4+ T Cells

**DOI:** 10.1371/journal.pone.0132957

**Published:** 2015-07-23

**Authors:** Ingvild S. Leikfoss, Pankaj K. Keshari, Marte W. Gustavsen, Anja Bjølgerud, Ina S. Brorson, Elisabeth G. Celius, Anne Spurkland, Steffan D. Bos, Hanne F. Harbo, Tone Berge

**Affiliations:** 1 Department of Neurology, Oslo University Hospital, Oslo, Norway; 2 Institute of Clinical Medicine, University of Oslo, Oslo, Norway; 3 Institute of Basic Medical Sciences, University of Oslo, Oslo, Norway; University of Tokyo, JAPAN

## Abstract

For multiple sclerosis, genome wide association studies and follow up studies have identified susceptibility single nucleotide polymorphisms located in or near *CLEC16A* at chromosome 16p13.13, encompassing among others *CIITA*, *DEXI* and *SOCS1* in addition to *CLEC16A*. These genetic variants are located in intronic or intergenic regions and display strong linkage disequilibrium with each other, complicating the understanding of their functional contribution and the identification of the direct causal variant(s). Previous studies have shown that multiple sclerosis-associated risk variants in *CLEC16A* act as expression quantitative trait loci for *CLEC16A* itself in human pancreatic β-cells, for *DEXI* and *SOCS1* in thymic tissue samples, and for *DEXI* in monocytes and lymphoblastoid cell lines. Since T cells are major players in multiple sclerosis pathogenesis, we have performed expression analyses of the *CIITA-DEXI-CLEC16A-SOCS1* gene cluster in CD4+ and CD8+ T cells isolated from multiple sclerosis patients and healthy controls. We observed a higher expression of *SOCS1* and *CLEC16A* in CD4+ T cells in samples homozygous for the risk allele of *CLEC16A* rs12927355. Pair-wise linear regression analysis revealed high correlation in gene expression in peripheral T cells of *CIITA*, *DEXI*, *CLEC16A* and *SOCS1*. Our data imply a possible regulatory role for the multiple sclerosis-associated rs12927355 in *CLEC16A*.

## Introduction

Multiple sclerosis (MS) is a chronic inflammatory demyelinating disease of the central nervous system [1]. The cause of MS is not completely understood, however, both environmental and genetic factors contribute to disease risk [2, 3]. In addition to HLA-DRB1*15:01, which is the strongest genetic risk allele in MS, 110 non-HLA MS risk variants have been identified [4]. A single nucleotide polymorphism (SNP) in the C-type lectin like domain family 16, member A (*CLEC16A*) gene was among the first genetic variants outside the HLA-region that showed suggestive association in the first genome-wide association study (GWAS) on MS [5]. SNPs in *CLEC16A* have since then been convincingly replicated in MS studies [6, 7]. Although there are additional independent genetic signals from SNPs located in the 16p13.13 chromosomal region, such as in the *CLEC16A*-*SOCS1* intergenic region [8], in *CIITA* [9, 10] and in *SOCS1* [11], *CLEC16A* has been suggested to be the most likely causal gene in this region as it contains the strongest MS-associated SNPs [4, 8]. In addition to MS, SNPs in *CLEC16A* have been shown to be associated with several other autoimmune diseases, as reviewed in [7], including type 1 diabetes (T1D), Crohn`s disease, Addison’s disease and rheumatoid arthritis. Disease-associated SNPs in *CLEC16A* are mainly located in intronic regions and display strong linkage disequilibrium (LD), making it difficult to comprehend their independent functions or identify the direct causal variant(s). Non-coding disease-associated SNPs may contribute to disease by acting as expression quantitative trait loci (eQTL). In a previous report, we showed that the expression of *DEXI* and *SOCS1* in human thymic tissue samples was associated with the genotype of *CLEC16A* SNPs [12] that displayed the strongest association with MS in a combined British and Norwegian cohort [13]. The top-hit from that screen, rs12708716, is in strong LD (r^2^ = 0.82, D’ = 1.00) with the *CLEC16A* SNP rs12927355, which is the primary SNP at this locus identified through a large-scale consortium based analysis using the ImmunoChip [4]. In addition, others have shown association of rs12708716 with *DEXI* expression in monocytes [14] and B lymphoblastoid cell lines [15] and with the expression of *CLEC16A* itself in human pancreatic β-cells [16]. Taken together, this indicates that this intronic *CLEC16A* SNP represent eQTLs for at least three of the genes in this region, i.e. *CLEC16A*, *DEXI* and *SOCS1*.

Both *CIITA* and *SOCS1* are compelling candidate genes for autoimmune diseases [11, 17–21] as their functions in immune cells are well established. *CIITA* encodes the MHC class II transactivator, which is a co-regulator of MHC class II gene expression [22], whereas the protein encoded by *SOCS1* is a negative regulator of cytokine signaling important for immune cell homeostasis and regulation of inflammation [23]. Although CLEC16A has been implicated in endosomal transport and autophagy in *Drosophila melanogaster [24, 25]*, mitophagy in murine β cells [16], B cell development in a *Clec16a* knock-down mouse model [26] and late endosome biogenesis and HLA class II expression in human antigen-presenting cells (APCs) [27], its function in human T cells is poorly understood. *DEXI*, a dexamethasone induced gene, encodes a protein with unknown function [28].

T cells are major players in MS pathogenesis [29], and a recent study showed that SNPs associated with MS and other autoimmune diseases preferentially map to enhancers and promoters active in T cell subsets [30], indicating that these cells are indeed relevant for eQTL studies of MS-associated SNPs. We have analyzed the gene expression of *CIITA*, *DEXI*, *CLEC16A* and *SOCS1* in peripheral CD4+ and CD8+ T cells obtained from MS patients and healthy controls (HCs). First, we compared the overall expression of these genes between MS patients and controls. Thereafter, the expression of these genes was tested for association with the primary and secondary MS-associated *CLEC16A* SNPs reported by the ImmunoChip study, rs12927355 and rs4780346, respectively [4]. Furthermore, since pair-wise co-expression of several of the *CIITA*, *DEXI*, *CLEC16A* and *SOCS1* genes have been observed in thymic tissue samples [12], in human lymphoblastoid cell lines [8] and in whole blood [31], we aimed to determine whether this co-expression persisted in peripheral T cells.

## Materials and Methods

### Subjects and genotyping

A collection of 33 untreated, female Norwegian MS patients with relapsing remitting MS (RRMS) and 29 age-matched female HCs were included. All patients and controls were of Nordic ancestry. Patients were recruited from the MS clinic at the Oslo University Hospital, Oslo, Norway and controls either through the patients or among hospital employees (Table 1). None of the patients had ever received immune-modulatory drugs except steroids. Patients had not experienced a relapse or received steroids in the three months prior to enrolment and fulfilled the revised McDonald criteria [32]. The Regional Committee for Medical and Health Research Ethics South East, Norway, approved this study. Written informed consent was obtained from all study participants. Genome-wide SNP genotypes for patients and controls were assessed using the Human Omni Express BeadChip (Illumina, San Diego, CA, USA) as described previously [33]. We obtained genotypes for two SNPs in *CLEC16A*, rs2041670 and rs7203535, which are in full LD (r^2^ = 1.00, D` = 1.00) with the ImmunoChip hits rs12927355 and rs4780346, respectively, for all except four samples, which were excluded from the expression analyses of samples grouped based on genotype. We will refer to the ImmunoChip SNP IDs throughout the paper.

**Table 1:** Characteristics of MS patients and controls.

	Age^1^	Age at onset	Years MS^1^	EDSS^1^
Patients				
Mean (S.D.; range)	39.5 (9.2; 21–63)	29.75 (7.4; 19–34)	9.6 (9.2; 0–33)	2,0 (1.5; 0–6)
Controls				
Mean (S.D.; range)	39.6 (8.9; 22–58)	N/A	N/A	N/A

^1^ At inclusion in this study.

Abbreviations: EDSS = expanded disability status scale, S.D. = standard deviation, N/A = not applicable.

### Sample collection

CD4+ T cells and CD8+ T cells were isolated from whole blood from MS patients and healthy controls as described previously [33]. Briefly, 64 ml of whole blood was collected in EDTA coated vacuum tubes (Greiner Bio-One, Frickenhausen, Germany). Peripheral blood mononuclear cells (PBMC) were separated from EDTA-blood, washed and resuspended in ice cold PBS (Life Technologies, Paisley, UK) following centrifugation. CD8+ microBeads (Miltenyi Biotec, Lund, Sweden) were added to PBMCs and positively isolated using autoMACS cell separator (Miltenyi Biotec) and a positive selection program. CD4+ T cells were then isolated from the negative fraction using CD4+ negative selection microBeads (Miltenyi Biotec) and the negative selection program. Cell purity was assessed by flow cytometry as described [33], and the CD4+ and CD8+ T cell fractions were > 95% pure. For each cell type, 2x10^6^ cells were stored in RNA protect (Qiagen, Hilden, Germany) at -80°C.

### Isolation of RNA, cDNA synthesis and gene expression analysis

RNA from CD4+ T cells (RRMS = 28, HC = 26,) and CD8+ T cells (RRMS = 17, HC = 23) stored on RNA protect was isolated using RNeasy Mini Kit and Qiashredder spin columns (Qiagen). The RNA concentration, quality and integrity were measured by Nanodrop 2000c spectrophotometer (Thermo Fisher Scientific Inc., Madison, WI, USA) and Agilent 2100 Bioanalyzer (Agilent Technologies, Santa Clara, CA, USA). 200 ng RNA was reverse transcribed (RT) to cDNA using the Maxima First Strand cDNA synthesis Kit (Thermo Scientific) in a 20 μl reaction. RT was performed using the GeneAmp PCR system 9700 thermo cycler (Applied Biosystems) for a one-step PCR (25°C for 10 min, 48°C for 30 min and 95°C for 5 min) as per manufactures instructions. cDNA was diluted 9 fold in RNase-free water (Qiagen) prior to the real time PCR reaction, to a final concentration of 1.11 ng/μl. A standard curve was prepared from PBMCs from healthy donors as a 1:3-fold dilution series (50–0.20 ng/μl). The quantitative real-time PCR (qPCR) was performed in 10 μl final volume containing 0.5 μl of 20x Primer Probe (as specified below; Applied Biosystems), 0.96 ng cDNA, 5 μl TaqMan Gene Expression Mastermix (Applied Biosystems) and 4 μl RNase-free water (Qiagen). The PCR plate included a negative control without cDNA and a no-RT control. Primers and probes against *TBP* (4326322E), *18S rRNA* (4319413E-1006049), *SDHA* (Hs00188166_m1), total *CLEC16A* (HS00389799_m1), *DEXI* (HS00360234_m1), total *CIITA* (HS00172094_m1) or *SOCS1* (HS00705164_s1) (all from Applied Biosystems) were added to each reaction. For the genes with more than one transcript, the assay covering most transcripts was selected. QPCR amplification was performed using the ViiA7 Real-Time PCR system (Applied Biosystem). The samples were run in duplicates on a MicroAmp Optical 384 well reaction plate (Applied Biosystems) and analyzed by sequence detection systems (SDS) v. 2.3 (Applied Biosystems) relative to three reference genes. *TBP* was selected as the preferential reference gene given its low variance in C_T_ between the different samples (data not shown). PCR specificity was confirmed by a single band after agarose gel electrophoresis.

### Statistical analysis

A Mann—Whitney U test was performed to compare gene expression levels between MS patients versus controls and for the gene expression in relation to genotypes (creating pools for carriers of the minor alleles). Pair-wise linear regression analysis was used to obtain coefficient of determination (r^2^) for the gene expression between *CIITA*, *DEXI*, *CLEC16A* and *SOCS1*. All statistical analyses were performed using GraphPad Prism 6 (GraphPad software, Inc., San Diego, CA, USA).

## Results

### No difference in gene expression between MS patients and healthy controls

**Fig 1:**
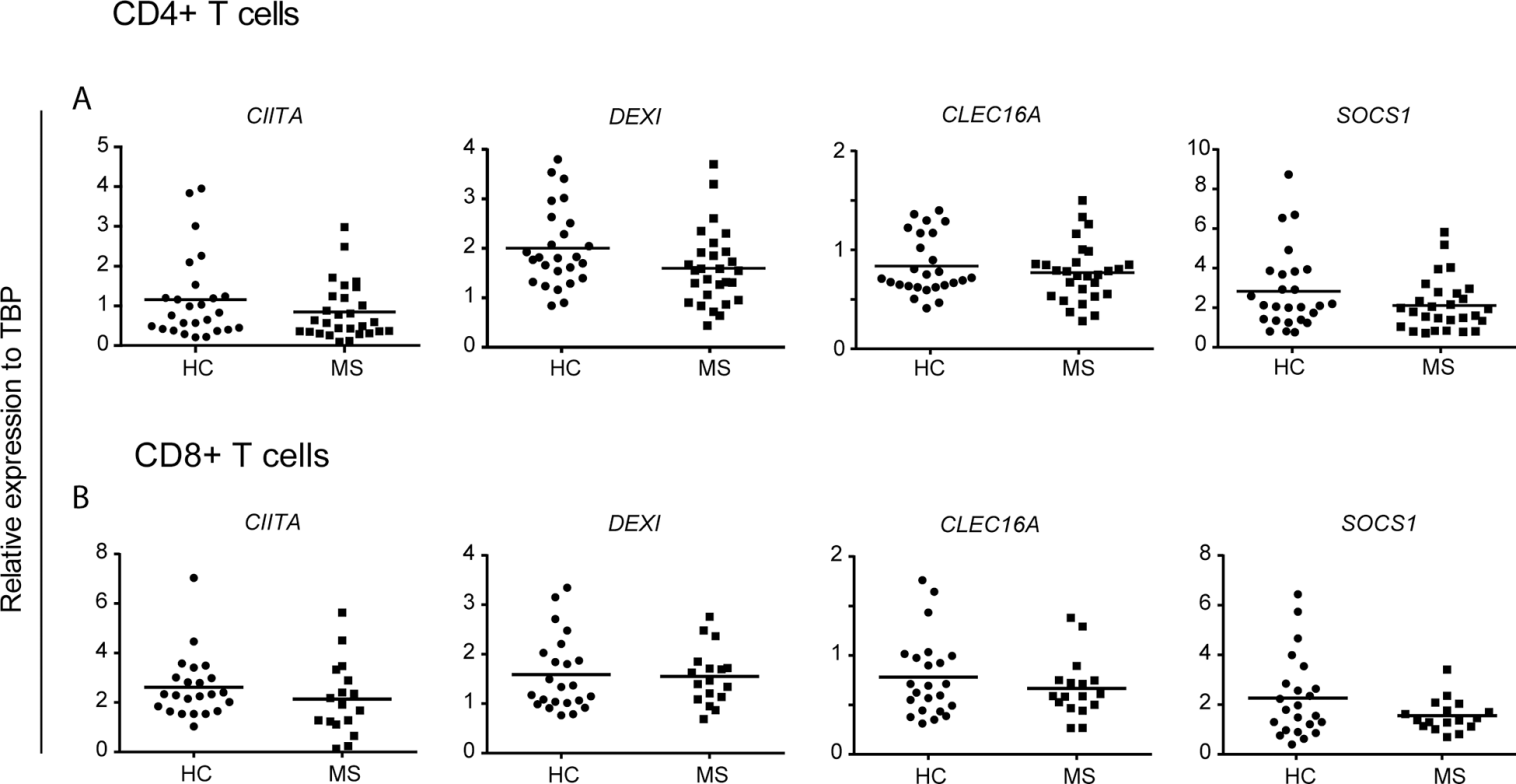
No difference in 16p13.13 T cell expression between MS patients and healthy controls. The plots show gene expression of *CIITA*, *DEXI*, *CLEC16A* and *SOCS1* relative to *TBP* in (A) CD4+ T cells (MS: n = 28; HC: n = 26) and (B) CD8+ T cells (MS n = 17; HC: n = 23). Mann-Whitney U-test was performed to compare the groups. The median value in each group is indicated as a horizontal line.

The risk locus containing *CLEC16A* is a well-established susceptibility gene region for autoimmune diseases, including MS. We have previously analyzed gene expression of *CLEC16A* and the surrounding genes, *CIITA*, *DEXI* and *SOCS1* (S1 Fig), in whole blood and thymic tissue samples [12, 13]. T cells are major players in the development of MS [29]. Genetic data further indicate that an enrichment of MS risk loci is identified in DNase hypersensitive sites (DHSs), i.e. associated with active transcription, in cell types of relevance for the MS disease, among them CD4+ and CD8+ T cells [34, 35]. We first set out to measure gene expression of *CLEC16A* and the surrounding genes, *CIITA*, *DEXI* and *SOCS1*, in peripheral T cells purified from treatment-naïve, female RRMS patients and age- and sex-matched healthy controls (Table 1). We did not observe any significant differences in gene expression between MS patients and controls for any of the four genes in CD4+ (Fig 1A) or in CD8+ T cells (Fig 1B). When subdividing the samples from the MS patients based on disease duration, we did not observe any differences in 16p13.13 gene expression between patients who had MS for several years (8–33 years) compared to patients who were relatively newly diagnosed with MS (0–3 years) (data not shown).

### *CLEC16A*, *DEXI* and *SOCS1* expression is affected by *CLEC16A* genotype

*CLEC16A* SNPs have been suggested to act as eQTLs for the genes in the *CIITA*-*DEXI*-*CLEC16A*-*SOCS1* gene complex [8, 12, 14, 15, 36]. We analyzed whether the ImmunoChip *CLEC16A* hits had an impact on the expression of the genes encoded at this locus in CD4+ and CD8+ T cells. Since we did not observe any differences in gene expression between MS patients and controls for those genes, samples were pooled by carriers of the minor allele for rs12927355 (minor allele frequency; MAF = 0.275, minor allele = A) and rs4780346 (MAF = 0.325, minor allele = A), the primary and secondary ImmunoChip signals, respectively [4].

**Fig 2:**
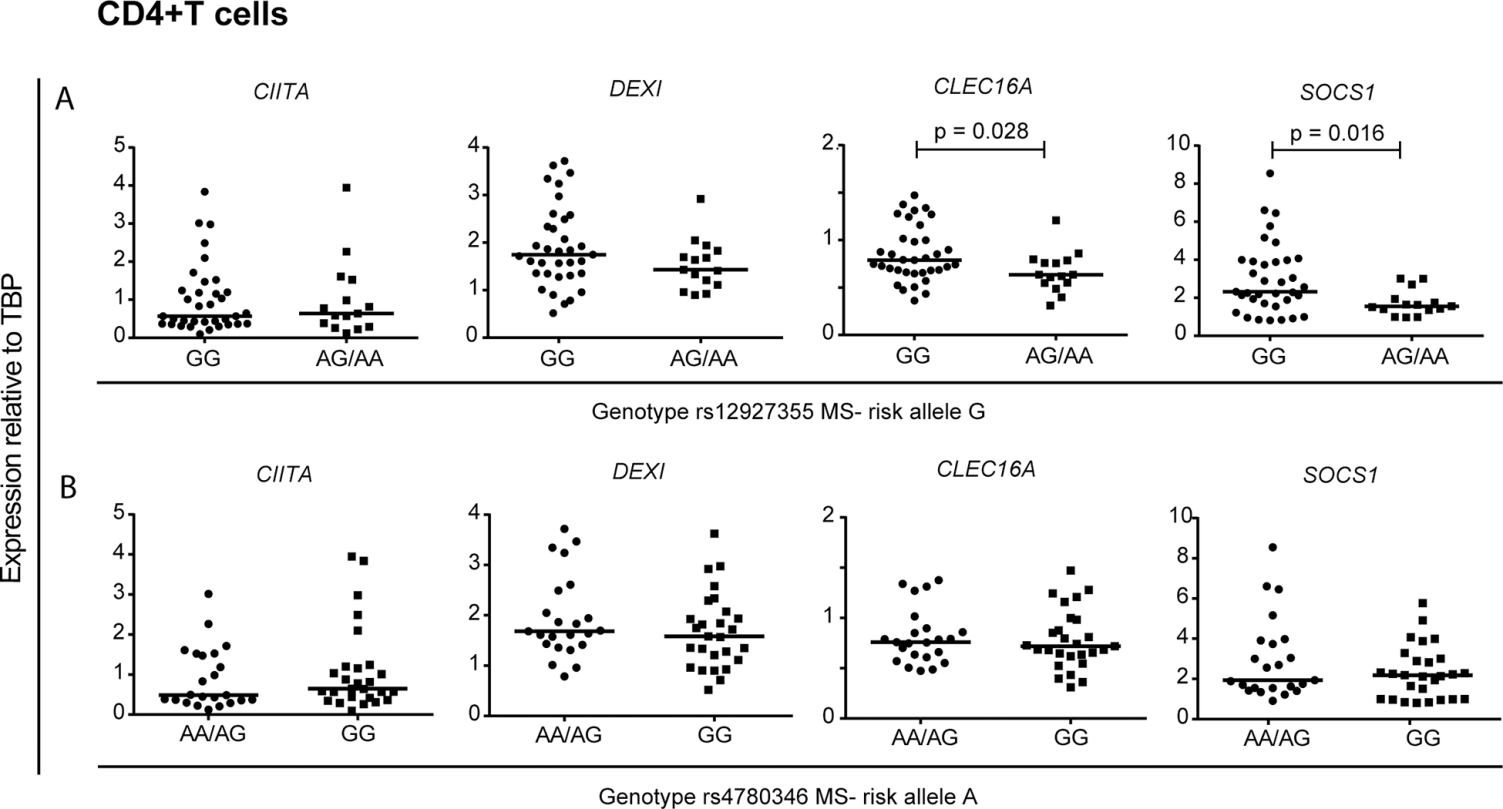
The genotype of rs12927355 associates with increased expression of *CLEC16A* and *SOCS1* in CD4+T cells. The plots show gene expression of *CIITA*, *DEXI*, *CLEC16A* and *SOCS1* relative to *TBP* in CD4+ T cells (n = 50) from MS patients (n = 27) and HCs (n = 23). The samples were sorted according to *CLEC16A* genotype of two MS-associated SNPs (A) rs12927355 (risk allele = G): GG: n = 35, AG: n = 14 and AA: n = 1, and (B) rs4780346 (risk allele = A): AA: n = 4 and AG: n = 19, GG: n = 27. Mann-Whitney U-test was performed to compare the groups. Significant *P*-values are shown in the figure. The median value in each group is indicated as a horizontal line.

**Fig 3:**
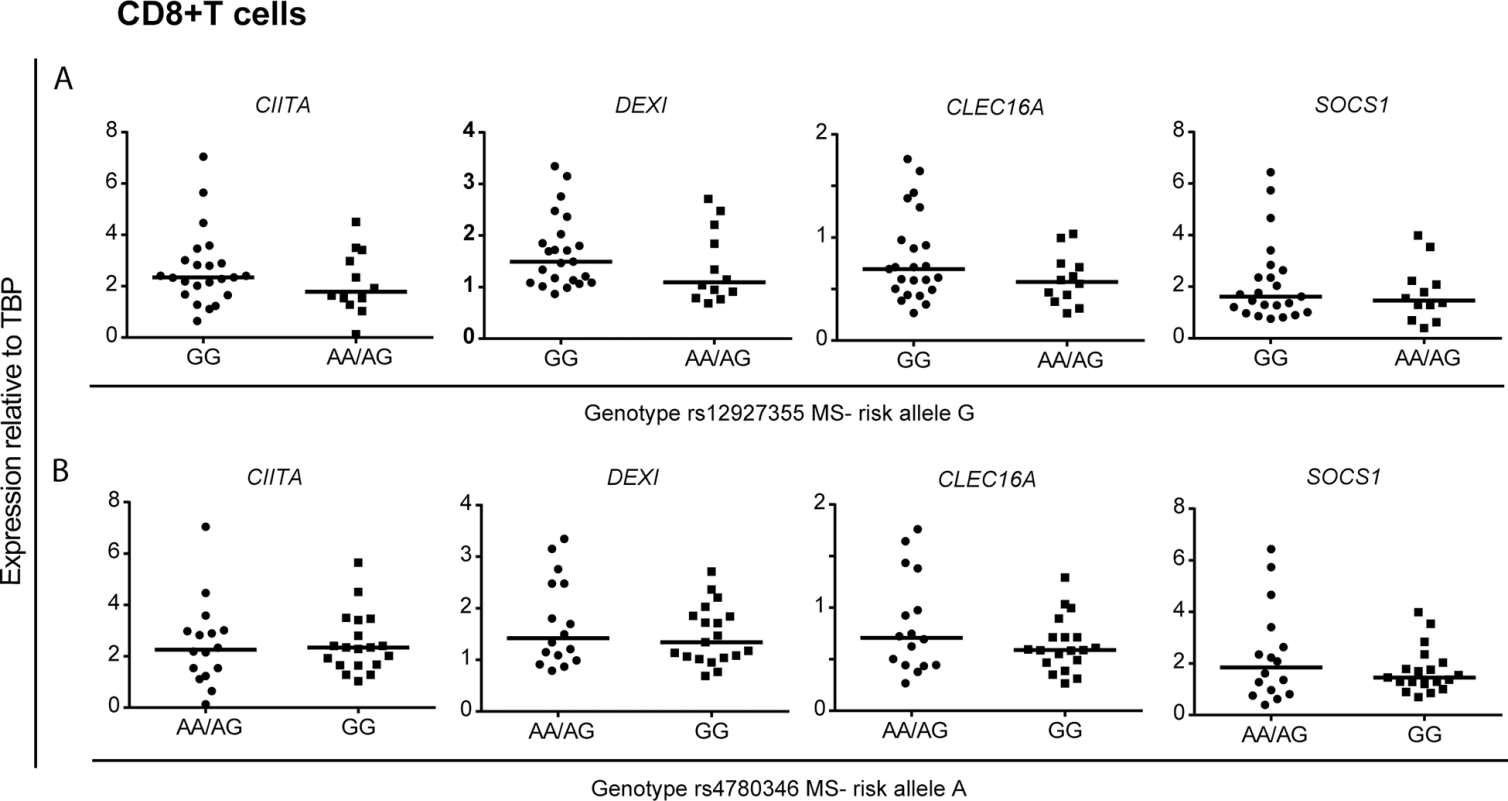
No association with *CLEC16A* MS risk SNPs and 16p13.13 gene expression in CD8+ T cells. The plots show gene expression of *CIITA*, *DEXI*, *CLEC16A* and *SOCS1* relative to *TBP* in CD8+ T cells (n = 35) from MS patients (n = 15) and HCs (n = 20). The samples were sorted according to *CLEC16A* genotype of two MS-associated SNPs (A) rs12927355 (risk allele = G): GG: n = 23, AG: n = 10 and AA: n = 2, and (B) rs4780346 (risk allele = A): AA: n = 1 and AG: n = 15, GG: n = 19. Mann-Whitney U-test was performed to compare the groups. The median value in each group is indicated as a horizontal line.

In CD4+ T cells, we observed a significantly higher *SOCS1* and *CLEC16A* expression in the samples homozygous for the rs12927355 risk allele (GG) compared to the samples carrying the non-risk allele (AG/AA; Fig 2A), whereas we observed no differences in gene expression in samples sorted for the genotype of rs4780346 in these cells (Fig 2B). We did not observe any significant association between gene expression of *CIITA*, *DEXI*, *CLEC16A* or *SOCS1* and the two SNPs in CD8+ T cells (Fig 3).

### *CIITA*, *DEXI*, *CLEC16A* and *SOCS1* are co-expressed in human T cells

We and others have previously observed an association between *CLEC16A* expression and that of *SOCS1* and *DEXI* in thymic tissue samples [12] and lymphoblastoid cell lines [8], but not in whole blood [12] nor in monocytes [14]. Since approximately 30% of all genes show discordant tissue-dependent regulation [37], we investigated whether there was any correlation in gene expression between *CIITA*, *DEXI*, *CLEC16A* and *SOCS1* in peripheral CD4+ and CD8+ T cells. We performed a pair-wise linear regression analysis between the expression of the genes for both CD4+ and CD8+ T cells (Table 2). A high correlation was observed between all four genes in CD4+ T cells, with especially high correlation between *DEXI* and *SOCS1* expression (r^2^ = 0.62, *P* < 0.0001). Additionally, all gene combinations except *CIITA* and *DEXI* showed high correlation in gene expression in CD8+ T cells (Table 2).

**Table 2:** Pair-wise linear regression analysis of *CIITA*, *DEXI*, *SOCS1* and *CLEC16A* expression in CD4+ and CD8+ T cells.

		*CIITA*	*DEXI*	*SOCS1*
	*DEXI*	r^2^ = 0.1472, *P* = 0.0018*		
CD4+	*SOCS1*	r^2^ = 0.1016, *P* = 0.0188*	r^2^ = 0.6188, *P* < 0.0001*	
	*CLEC16A*	r^2^ = 0.0923, *P* = 0.0256*	r^2^ = 0.1486, *P* = 0.004*	r^2^ = 0.1770, *P* = 0.0015*
		*CIITA*	*DEXI*	*SOCS1*
	*DEXI*	r^2^ = 0.0907, *P* = 0.059		
CD8+	*SOCS1*	r^2^ = 0.2370, *P* = 0.0014*	r^2^ = 0.7191, *P* < 0.0001*	
	*CLEC16A*	r^2^ = 0.2493, *P* = 0.001*	r^2^ = 0.7677, *P* < 0.0001*	r^2^ = 0.7995, *P* < 0.0001*

r^2^ represents the coefficient of determination and *P* is uncorrected *P* value.

* represents significant correlations.

## Discussion

GWASs have identified several loci associated with autoimmune diseases, however, the causal variants remain largely unknown [30]. In MS, samples were typed on a genotyping platform (ImmunoChip) designed to deeply interrogate 184 non-MHC loci with genome-wide significant associations in at least one autoimmune disease. This study identified rs12927355 as the primary signal within *CLEC16A* [4]. Here we report that the genotype of rs12927355 (intron 19 of *CLEC16A*) associates with gene expression of *CLEC16A* and *SOCS1* in human peripheral CD4+ T cells. Furthermore, we show that the four studied genes, i.e. *CIITA*, *DEXI*, *CLEC16A* and *SOCS1*, are co-expressed in peripheral CD4+ and CD8+ T cells.

Several GWASs have identified SNPs in the *CIITA-DEXI-CLEC16A-SOCS1* gene cluster on chromosome 16p13.13, as associated with autoimmune diseases [4] (reviewed in [7]). In human islet cells, increased *CLEC16A* expression was associated with the MS risk variant at rs12708716 [16], while we previously observed that this SNP was associated with reduced *SOCS1* expression in thymic tissue samples, but had no impact on *CIITA*, *DEXI* or *CLEC16A* expression [12]. Recently, stratification according to the risk SNP rs7200786 in LD with rs12708716 and rs12927355 (both r^2^ = 0.61, D’ = 1) revealed no effect on *CLEC16A* expression in blood. However when correcting for immune cell frequencies in blood, a weak correlation was found with CD4+ T cells in samples from MS cases [27]. When analyzing gene expression of *CLEC16A*, *DEXI* and *SOCS1* in whole blood from common variable immunodeficiency patients, higher level of *CLEC16A* expression was only observed in the AA group (homozygous for the protective allele) of rs17806056, also in partial LD with rs12708716 (r^2^ = 0.555) [26]. Our current finding in CD4+ T cells where higher *CLEC16A* and *SOCS1* expression was associated with the MS-risk allele at rs12927355 partially supports these previous observations. This SNP is in strong LD with rs12708716 (r^2^ = 0.82, D` = 1.00). In addition, both rs12927355 and rs12708716 are located in active regions with H3K27 acetylation, a marker for active enhancers [38]. However, we cannot exclude the possibility that another causal variant in LD with those SNPs might have effects on the expression of 16p13.13 genes in CD4+ T cells as well as in other cell types. In fact, for most of the SNPs that have been shown to be associated with complex diseases, the underlying SNP is predicted to be located within the LD block of the associated SNP [30].

For the secondary ImmunoChip signal, rs4780346, we did not observe any association with gene expression for any of the four genes, neither in CD4+ nor in CD8+ T cells. This SNP is in partial linkage with the primary ImmunoChip SNP, rs12927355 (r^2^ = 0.18, D` = 1.00), indicating that the significant changes in *CLEC16A* and *SOCS1* expression observed for rs12927355 are likely not attributable to functional properties of rs4780346 in these cell types. In contrast to the CD4+ T cells, we did not observe any genotype dependent expression differences for either of the SNPs in CD8+ T cells. This is in line with the pathway analyses of MS associated loci, identifying an overrepresentation of genes involved in T helper cell differentiation [6]. On the other hand, another study showed that MS associated SNPs overlap with immune-specific DHSs more than expected by chance, especially DHSs from T cell subsets including CD8+ T cells and Th1 and Th17 CD4+ T cells [34]. The reason for the lack of association between genotype and gene expression could be due to the smaller sample size of CD8+ T cells compared to CD4+ T cells, or due to cell-specific differences in gene regulation, where the causal SNP(s) in this region might affect binding of CD4+ T-cell specific transcription factors.

Different cell types have different epigenetic profiles [39] and can give rise to the observed gene expression differences described above. As epigenetics is changed by aging and hormones [40–42] it might influence gene expression differently in the cohorts. For instance, the thymic tissue samples were collected from young children of both sexes undergoing cardiac surgery [12, 13], whereas the CD4+ and CD8+ T cells in our study were isolated from women aged 21–63 and the islets were isolated from non-diabetic cadaver donors [16]. Thus, the 16p13.13 gene expression differences observed between different tissues could be explained by differences in accessibility of promoters and enhancers in the different cell types, or by cell-type dependent eQTLs [37, 43]. Whether *CLEC16A* genotype affects expression of these 16p13.13 genes in other immune cells or in subtypes of the CD4+ and CD8+ T cell lineages remains to be studied.

A pair-wise linear regression analysis of the four genes studied showed that their expression was highly correlated in peripheral T cells, with the exception of *CIITA* and *DEXI* in CD8+ T cells. For three of the genes, this is supported by previous findings in human thymic tissue samples [12] as well as in human lymphoblastoid cell lines [8], where *CLEC16A*, *SOCS1* and *DEXI* were shown to be co-expressed. This correlation in gene expression was not observed in whole blood [12] nor in monocytes [14], indicating different expression patterns in different cell types.

We did not observe differences in gene expression of *CIITA*, *DEXI*, *CLEC16A* or *SOCS1* in T cells between MS patients and controls, indicating that the MS disease itself does not impact the expression of these genes in the studied cells. Of note, our cohort of untreated MS patients are either recently diagnosed with MS or have a benign disease course. It remains uncertain whether possible differences in gene expression in T cells from patients and controls could have been detected at a different stage of the disease, as gene expression changes over time or can be altered by factors such as inflammation and oxidative stress [44–46].

Whether the genotype-dependent association with *CLEC16A* and *SOCS1* expression also exists at the protein level remains to be studied. Based on studies from other immune cells, altered *CLEC16A* expression affects antigen presentation and HLA-II expression in APCs [27] and T cell selection due to an effect of CLEC16A on autophagy in murine thymic epithelial cells [47]. The biological function of *CLEC16A* in T cells has so far not been assessed. Thus, further studies to explore the impact of *CLEC16A* on the T cell phenotype are necessary. SOCS1 act as a negative regulator of cytokine signaling by regulating the JAK-STAT pathway [48]. T and NK cells from *Socs1* knockout mice produce more IFNγ and show resistance to Th17 dependent autoimmunity due to reduced Th17 cell differentiation [49]. Consequently, increased *SOCS1* expression in T cells might be unfavorable in relation to autoimmunity as a result of an increased Th17 inflammatory profile. An increase in the CD4+/CD8+ T cell ratio has been observed in peripheral blood from MS patients compared to controls [50]. Since homozygosity for the risk allele for rs12927355 correlates with increased *CLEC16A* and *SOCS1* expression in CD4+ T cells, but has no significant impact in CD8+ T cells, the increase of the CD4+/CD8+ T cell ratio in MS patients would lead to an even higher total T cell expression of *CLEC16A* and *SOCS1* in MS patients homozygous for the risk allele compared to healthy controls with the same genotype. We do not know what impact this has for our cohort of MS patients and healthy controls as we have not measured the CD4+/CD8+ T cell ratio prior to cell purification. In the current study, we showed that the genotype of rs12927355 has functional impact in CD4+ T cells. Further studies regarding the functional implications of this regulatory region, and a more detailed investigation into the putative roles in immune homeostasis of the different genes harbored in this region in immune cell subsets are warranted to understand the role of these genes in autoimmune disease.

## Supporting Information

S1 Fig Schematic drawing of the *CIITA*-*DEXI-CLEC16A-SOCS1* gene complex on chromosome 16p13.13. The primary ImmunoChip SNPs in *CLEC16A*, rs12927355, is located in intron 19, while rs4780346, the secondary immunoChip SNP is located in the *CLEC16A*-*SOCS1* intergenic region.
(TIF)

## References

[1] Compston A, Coles A. Multiple sclerosis. Lancet. 2008;372(9648):1502–17. 10.1016/S0140-6736(08)61620-7 . 18970977

[2] O'Gorman C, Lucas R, Taylor B. Environmental risk factors for multiple sclerosis: a review with a focus on molecular mechanisms. Int J Mol Sci. 2012;13(9):11718–52. 10.3390/ijms130911718 23109880 PMC3472772

[3] Sawcer S, Franklin RJ, Ban M. Multiple sclerosis genetics. Lancet Neurol. 2014;13(7):700–9. 10.1016/S1474-4422(14)70041-9 . 24852507

[4] International Multiple Sclerosis Genetics C, Beecham AH, Patsopoulos NA, Xifara DK, Davis MF, Kemppinen A, et al Analysis of immune-related loci identifies 48 new susceptibility variants for multiple sclerosis. Nature genetics. 2013;45(11):1353–60. 10.1038/ng.2770 24076602 PMC3832895

[5] International Multiple Sclerosis Genetics C, Hafler DA, Compston A, Sawcer S, Lander ES, Daly MJ, et al Risk alleles for multiple sclerosis identified by a genomewide study. N Engl J Med. 2007;357(9):851–62. 10.1056/NEJMoa073493 . 17660530

[6] International Multiple Sclerosis Genetics C, Wellcome Trust Case Control C, Sawcer S, Hellenthal G, Pirinen M, Spencer CC, et al Genetic risk and a primary role for cell-mediated immune mechanisms in multiple sclerosis. Nature. 2011;476(7359):214–9. 10.1038/nature10251 21833088 PMC3182531

[7] Berge T, Leikfoss IS, Harbo HF. From Identification to Characterization of the Multiple Sclerosis Susceptibility Gene CLEC16A. Int J Mol Sci. 2013;14(3):4476–97. 10.3390/ijms14034476 23439554 PMC3634488

[8] Zuvich RL, Bush WS, McCauley JL, Beecham AH, De Jager PL, International Multiple Sclerosis Genetics C, et al Interrogating the complex role of chromosome 16p13.13 in multiple sclerosis susceptibility: independent genetic signals in the CIITA-CLEC16A-SOCS1 gene complex. Human molecular genetics. 2011;20(17):3517–24. 10.1093/hmg/ddr250 21653641 PMC3153306

[9] Gyllenberg A, Piehl F, Alfredsson L, Hillert J, Bomfim IL, Padyukov L, et al Variability in the CIITA gene interacts with HLA in multiple sclerosis. Genes Immun. 2014;15(3):162–7. 10.1038/gene.2013.71 . 24430172

[10] Bronson PG, Caillier S, Ramsay PP, McCauley JL, Zuvich RL, De Jager PL, et al CIITA variation in the presence of HLA-DRB1*1501 increases risk for multiple sclerosis. Human molecular genetics. 2010;19(11):2331–40. 10.1093/hmg/ddq101 20211854 PMC2865376

[11] Vandenbroeck K, Alvarez J, Swaminathan B, Alloza I, Matesanz F, Urcelay E, et al A cytokine gene screen uncovers SOCS1 as genetic risk factor for multiple sclerosis. Genes Immun. 2012;13(1):21–8. 10.1038/gene.2011.44 . 21716315

[12] Leikfoss IS, Mero IL, Dahle MK, Lie BA, Harbo HF, Spurkland A, et al Multiple sclerosis-associated single-nucleotide polymorphisms in CLEC16A correlate with reduced SOCS1 and DEXI expression in the thymus. Genes Immun. 2013;14(1):62–6. 10.1038/gene.2012.52 . 23151489

[13] Mero IL, Ban M, Lorentzen AR, Smestad C, Celius EG, Saether H, et al Exploring the CLEC16A gene reveals a MS-associated variant with correlation to the relative expression of CLEC16A isoforms in thymus. Genes Immun. 2011;12(3):191–8. 10.1038/gene.2010.59 . 21179112

[14] Davison LJ, Wallace C, Cooper JD, Cope NF, Wilson NK, Smyth DJ, et al Long-range DNA looping and gene expression analyses identify DEXI as an autoimmune disease candidate gene. Human molecular genetics. 2012;21(2):322–33. 10.1093/hmg/ddr468 21989056 PMC3276289

[15] Tomlinson MJt, Pitsillides A, Pickin R, Mika M, Keene K, Hou X, et al Fine Mapping and Functional Studies of Risk Variants for Type 1 Diabetes at Chromosome 16p13.13. Diabetes. 2014 10.2337/db13-1785 . 25008175 PMC4237999

[16] Soleimanpour SA, Gupta A, Bakay M, Ferrari AM, Groff DN, Fadista J, et al The diabetes susceptibility gene Clec16a regulates mitophagy. Cell. 2014;157(7):1577–90. 10.1016/j.cell.2014.05.016 24949970 PMC4184276

[17] Skinningsrud B, Husebye ES, Pearce SH, McDonald DO, Brandal K, Wolff AB, et al Polymorphisms in CLEC16A and CIITA at 16p13 are associated with primary adrenal insufficiency. J Clin Endocrinol Metab. 2008;93(9):3310–7. 10.1210/jc.2008-0821 . 18593762

[18] Dubois PC, Trynka G, Franke L, Hunt KA, Romanos J, Curtotti A, et al Multiple common variants for celiac disease influencing immune gene expression. Nature genetics. 2010;42(4):295–302. 10.1038/ng.543 20190752 PMC2847618

[19] Swanberg M, Lidman O, Padyukov L, Eriksson P, Akesson E, Jagodic M, et al MHC2TA is associated with differential MHC molecule expression and susceptibility to rheumatoid arthritis, multiple sclerosis and myocardial infarction. Nature genetics. 2005;37(5):486–94. 10.1038/ng1544 . 15821736

[20] Eike MC, Skinningsrud B, Ronninger M, Stormyr A, Kvien TK, Joner G, et al CIITA gene variants are associated with rheumatoid arthritis in Scandinavian populations. Genes Immun. 2012;13(5):431–6. 10.1038/gene.2012.11 . 22513452

[21] Gyllenberg A, Asad S, Piehl F, Swanberg M, Padyukov L, Van Yserloo B, et al Age-dependent variation of genotypes in MHC II transactivator gene (CIITA) in controls and association to type 1 diabetes. Genes Immun. 2012;13(8):632–40. 10.1038/gene.2012.44 . 23052709

[22] Chang CH, Flavell RA. Class II transactivator regulates the expression of multiple genes involved in antigen presentation. J Exp Med. 1995;181(2):765–7. 7836928 10.1084/jem.181.2.765 PMC2191893

[23] Fenner JE, Starr R, Cornish AL, Zhang JG, Metcalf D, Schreiber RD, et al Suppressor of cytokine signaling 1 regulates the immune response to infection by a unique inhibition of type I interferon activity. Nat Immunol. 2006;7(1):33–9. 10.1038/ni1287 . 16311601

[24] Kim S, Naylor SA, DiAntonio A. Drosophila Golgi membrane protein Ema promotes autophagosomal growth and function. Proc Natl Acad Sci U S A. 2012;109(18):E1072–81. 10.1073/pnas.1120320109 22493244 PMC3344964

[25] Kim S, Wairkar YP, Daniels RW, DiAntonio A. The novel endosomal membrane protein Ema interacts with the class C Vps-HOPS complex to promote endosomal maturation. J Cell Biol. 2010;188(5):717–34. 10.1083/jcb.200911126 20194640 PMC2835942

[26] Li J, Jorgensen SF, Maggadottir SM, Bakay M, Warnatz K, Glessner J, et al Association of CLEC16A with human common variable immunodeficiency disorder and role in murine B cells. Nature communications. 2015;6:6804 10.1038/ncomms7804 . 25891430 PMC4444044

[27] van Luijn MM, Kreft KL, Jongsma ML, Mes SW, Wierenga-Wolf AF, van Meurs M, et al Multiple sclerosis-associated CLEC16A controls HLA class II expression via late endosome biogenesis. Brain: a journal of neurology. 2015 10.1093/brain/awv080 . 25823473 PMC4614123

[28] Edgar AJ, Birks EJ, Yacoub MH, Polak JM. Cloning of dexamethasone-induced transcript: a novel glucocorticoid-induced gene that is upregulated in emphysema. Am J Respir Cell Mol Biol. 2001;25(1):119–24. 10.1165/ajrcmb.25.1.4417 . 11472984

[29] Hauser SL, Oksenberg JR. The neurobiology of multiple sclerosis: genes, inflammation, and neurodegeneration. Neuron. 2006;52(1):61–76. 10.1016/j.neuron.2006.09.011 . 17015227

[30] Farh KK, Marson A, Zhu J, Kleinewietfeld M, Housley WJ, Beik S, et al Genetic and epigenetic fine mapping of causal autoimmune disease variants. Nature. 2014 10.1038/nature13835 . 25363779 PMC4336207

[31] Lopez de Lapuente A, Pinto-Medel MJ, Astobiza I, Alloza I, Comabella M, Malhotra S, et al Cell-specific effects in different immune subsets associated with SOCS1 genotypes in multiple sclerosis. Mult Scler. 2015 10.1177/1352458514566418 . 25623250

[32] Polman CH, Reingold SC, Banwell B, Clanet M, Cohen JA, Filippi M, et al Diagnostic criteria for multiple sclerosis: 2010 revisions to the McDonald criteria. Ann Neurol. 2011;69(2):292–302. 10.1002/ana.22366 21387374 PMC3084507

[33] Bos SD, Page CM, Andreassen BK, Elboudwarej E, Gustavsen MW, Briggs F, et al Genome-Wide DNA Methylation Profiles Indicate CD8+ T Cell Hypermethylation in Multiple Sclerosis. PLoS One. 2015;10(3):e0117403 10.1371/journal.pone.0117403 . 25734800 PMC4348521

[34] Disanto G, Kjetil Sandve G, Ricigliano VA, Pakpoor J, Berlanga-Taylor AJ, Handel AE, et al DNase hypersensitive sites and association with multiple sclerosis. Human molecular genetics. 2014;23(4):942–8. 10.1093/hmg/ddt489 . 24092328

[35] Maurano MT, Humbert R, Rynes E, Thurman RE, Haugen E, Wang H, et al Systematic localization of common disease-associated variation in regulatory DNA. Science. 2012;337(6099):1190–5. 10.1126/science.1222794 22955828 PMC3771521

[36] Nica AC, Montgomery SB, Dimas AS, Stranger BE, Beazley C, Barroso I, et al Candidate causal regulatory effects by integration of expression QTLs with complex trait genetic associations. PLoS Genet. 2010;6(4):e1000895 10.1371/journal.pgen.1000895 20369022 PMC2848550

[37] Fu J, Wolfs MG, Deelen P, Westra HJ, Fehrmann RS, Te Meerman GJ, et al Unraveling the regulatory mechanisms underlying tissue-dependent genetic variation of gene expression. PLoS Genet. 2012;8(1):e1002431 10.1371/journal.pgen.1002431 22275870 PMC3261927

[38] Genome UCSC. UCSC Genome Browser 2002 [cited 2015 6 Feb]. Available from: www.genome.ucsc.edu.

[39] Slieker RC, Bos SD, Goeman JJ, Bovee JV, Talens RP, van der Breggen R, et al Identification and systematic annotation of tissue-specific differentially methylated regions using the Illumina 450k array. Epigenetics & chromatin. 2013;6(1):26 10.1186/1756-8935-6-26 23919675 PMC3750594

[40] Fraga MF, Esteller M. Epigenetics and aging: the targets and the marks. Trends Genet. 2007;23(8):413–8. 10.1016/j.tig.2007.05.008 . 17559965

[41] Fowden AL, Forhead AJ. Hormones as epigenetic signals in developmental programming. Exp Physiol. 2009;94(6):607–25. 10.1113/expphysiol.2008.046359 . 19251980

[42] Talens RP, Christensen K, Putter H, Willemsen G, Christiansen L, Kremer D, et al Epigenetic variation during the adult lifespan: cross-sectional and longitudinal data on monozygotic twin pairs. Aging cell. 2012;11(4):694–703. 10.1111/j.1474-9726.2012.00835.x 22621408 PMC3399918

[43] Dimas AS, Deutsch S, Stranger BE, Montgomery SB, Borel C, Attar-Cohen H, et al Common regulatory variation impacts gene expression in a cell type-dependent manner. Science. 2009;325(5945):1246–50. 10.1126/science.1174148 19644074 PMC2867218

[44] De Santis M, Selmi C. The Therapeutic Potential of Epigenetics in Autoimmune Diseases. Clinical Reviews in Allergy & Immunology. 2012;42(1):92–101. 10.1007/s12016-011-8293-8 WOS:000299000100010. 22161696

[45] Kunsch C, Medford RM. Oxidative stress as a regulator of gene expression in the vasculature. Circulation research. 1999;85(8):753–66. . 10521248 10.1161/01.res.85.8.753

[46] Bayarsaihan D. Epigenetic Mechanisms in Inflammation. J Dent Res. 2011;90(1):9–17. 10.1177/0022034510378683 21178119 PMC3144097

[47] Schuster C, Gerold KD, Schober K, Probst L, Boerner K, Kim MJ, et al The Autoimmunity-Associated Gene CLEC16A Modulates Thymic Epithelial Cell Autophagy and Alters T Cell Selection. Immunity. 2015;42(5):942–52. 10.1016/j.immuni.2015.04.011 25979422 PMC4439257

[48] Tamiya T, Kashiwagi I, Takahashi R, Yasukawa H, Yoshimura A. Suppressors of cytokine signaling (SOCS) proteins and JAK/STAT pathways: regulation of T-cell inflammation by SOCS1 and SOCS3. Arterioscler Thromb Vasc Biol. 2011;31(5):980–5. 10.1161/ATVBAHA.110.207464 . 21508344

[49] Tanaka K, Ichiyama K, Hashimoto M, Yoshida H, Takimoto T, Takaesu G, et al Loss of suppressor of cytokine signaling 1 in helper T cells leads to defective Th17 differentiation by enhancing antagonistic effects of IFN-gamma on STAT3 and Smads. J Immunol. 2008;180(6):3746–56. . 18322180 10.4049/jimmunol.180.6.3746

[50] Kolmel HW, Sudau C. T-cell subsets in the cerebrospinal fluid and blood of patients with multiple sclerosis. Journal of neuroimmunology. 1988;20(2–3):229–32. . 3264287 10.1016/0165-5728(88)90164-6

