# Supplementary figures and images for: Multiple Sclerosis Risk Allele in CLEC16A Acts as an Expression Quantitative Trait Locus for CLEC16A and SOCS1 in CD4+ T Cells
Source: PLoS One. 2015 Jul 23;10(7):e0132957. doi: 10.1371/journal.pone.0132957 (PMC4512731; doi:10.1371/journal.pone.0132957)

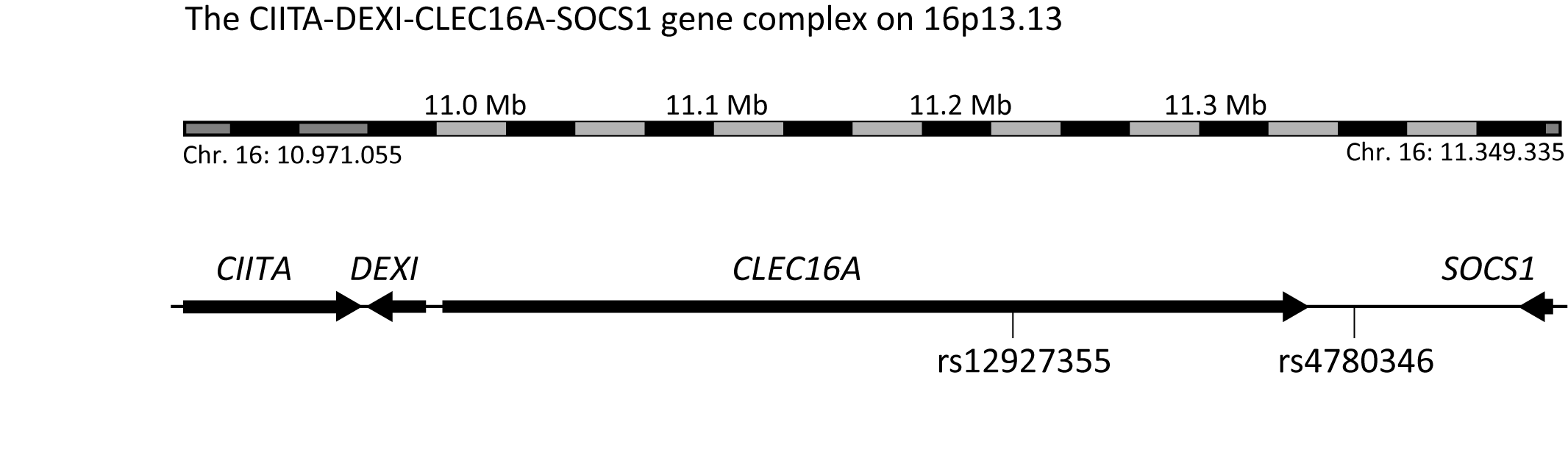

Supplement: S1 Fig — The primary ImmunoChip SNPs in CLEC16A, rs12927355, is located in intron 19, while rs4780346, the secondary immunoChip SNP is located in the CLEC16A-SOCS1 intergenic region. (TIF) [file pone.0132957.s001.tif]
